# Improving the reproducibility of the NAP1/B1/027 epidemic strain R20291 in the hamster model of infection

**DOI:** 10.1016/j.anaerobe.2016.02.011

**Published:** 2016-06

**Authors:** Michelle L. Kelly, Yen Kuan Ng, Stephen T. Cartman, Mark M. Collery, Alan Cockayne, Nigel P. Minton

**Affiliations:** a Clostridia Research Group, BBSRC/EPSRC Synthetic Biology Research Centre (SBRC), School of Life Sciences, Centre for Biomolecular Sciences, University of Nottingham, University Park, Nottingham, NG7 2RD, United Kingdom; b Nottingham Digestive Disease Centre, NIHR Biomedical Research Unit, The University of Nottingham, University Park, Nottingham, United Kingdom

**Keywords:** *Clostridium difficile*, Hamster model, Clindamycin sensitivity, *ermB*, Genome engineering

## Abstract

Comparative analysis of the *Clostridium difficile* BI/NAP1/027 strain R20291 and ClosTron-derived *ermB* mutants in the hamster infection model are compromised by the clindamycin susceptibility of the parent. Mutants can appear more virulent. We have rectified this anomaly by genome engineering. The variant created (CRG20291) represents an ideal control strain for virulence assays of ClosTron mutants.

*Clostridium difficile* is the leading cause of healthcare associated diarrhoea, causing almost half a million cases of *C. difficile* infection (CDI) in the United States in 2011 and 13,783 cases in the UK in 2014 [1], [2]. The situation has been exacerbated by the emergence of epidemic strains, and in particular BI/NAP1/027 strains typified by strain R20291 [3], [4]. Its use in the Golden Syrian hamster, the *in vivo* model of choice for infection studies [5], is complicated by the sensitivity of R20291 to clindamycin (Minimum Inhibitory Concentration, MIC = 16 μg/ml) [6]. Hamsters are dosed with clindamycin prior to infection with *C. difficile* to disrupt the normal gut flora. Clindamycin can persist at inhibitory levels (4–6 μg/g) some 11 days following administration [7], and has been shown to affect the time from infection to colonisation and death of strains with different clindamycin MICs [8]. One consequence is that ClosTron-generated mutants of R20291 in the hamster can show symptoms and succumb to disease earlier than those infected with the parent [9], a consequence of the introduction of the *ermB* gene during the mutagenesis.

A solution to the problem would be an *ermB* variant of R20291 which could be used as the isogenic, parental control in hamster assays of virulence that is otherwise unaffected in any other physiological characteristics. To create such a strain we synthesised the *ermB* gene of pMTL007C-E1 [10] such that it was immediately preceded by a sequence encompassing a clostridial thiolase gene promoter (P_thl_) and beginning with a NotI restriction site and was followed after its translational stop codon by a HindIII restriction site (see supplementary file). This appropriately cleaved DNA fragment was cloned between the NotI and HindIII restriction sites of the ACE (Allele-Coupled Exchange, [11]) complementation vector pMTL-YN2C [12] and the resultant plasmid (pMTL-YN2C:*ermB*) used to restore the previously made R20291 *pyrE* mutant (CRG2358) to prototrophy as previously described [12]. A DNA fragment from a randomly selected uracil prototrophic clone (designated CRG20291) was PCR amplified using appropriate primers (Table S1) and subjected to Sanger sequencing. This confirmed that the *ermB* gene and P_thl_ had inserted as intended, immediately downstream of the restored *pyrE* gene. Accordingly, CRG20291 was resistant to erythromycin (50 μg/ml). To determine the sensitivity to clindamycin, freshly grown colonies on BHIS (brain heart infusion supplemented with 0.5%, w/v, l-cysteine) agar were re-streaked on to BHIS medium supplemented with clindamycin. Plates were incubated anaerobically at 37 °C for 24 h and the MIC was designated as the lowest concentration where growth was inhibited. The MIC of CRG20191 had increased relative to R20291 (16 μg/ml) and was equivalent to that seen with the commonly studied strain 630 (Table 1).

To ascertain that CRG20291 was phenotypically indistinguishable from R20291, strains were cultured anaerobically at 37 °C in BHIS media and the colony forming units determined over a 72 h time period by plating appropriate dilutions onto BHIS agar. At the same time-points, toxin A and toxin B activity were measured by cell cytotoxicity assay [13] using Vero (African green monkey kidney) and HT29 (Human colon carcinoma) cell monolayers. No statistical difference was observed between either the growth of the two strains, or the amount of toxins produced (Fig. 1A). Similarly, using the sporulation assay of Burns et al. [14], no difference in the number of spores produced (data not shown) was evident following growth for 5 days on BHIS agar supplemented with 0.5% yeast extract (Sigma).

Finally, hamster infection studies were undertaken as previously described [9] in accordance with the UK Home Office Inspectorate under the Animals (Scientific Procedures) Act 1986 (see supplementary file for full details). Faecal and caecum samples collected during the course of the experiment were homogenised, heat treated and plated to look for the presence of *C. difficile*. To confirm the *C. difficile* isolated from these samples was the same strain originally used during infection, DNA was isolated from overnight cultures and PCR amplification was carried out using primer pairs specific to each host: 630 wild type: 4140 and 5880R; R20291: CDSM0-239-F1 and CDSM0-239-R1, and; CRG20291: Cdi-630-pyrD-sF1 and ermB-HindIII-R (see Table S1, for sequences).

Following infection with the R20291 parental strain of *C. difficile*, 75% of the animals infected were found to be colonised and all of these went on to show signs of infection, although the time from infection to clinical end point was spread between 52 and 83 h post infection (Fig. 1B). When strain 630 or CRG20291 were used for infection, 100% of the animals were found to be colonised and all went on to show signs of infection with 7 of the 8 animals reaching a clinical end point between either 44 and 72 h post infection (630) or 44 h post infection (CRG20291). The remaining animals went on to develop signs of infection and reached a clinical end point at either 91 h (630) or 76 h (CRG20291) post infection. The CRG20291 infected animals (Fig. 1B) reached the clinical defined end point 21.8 h earlier than the animals infected with R20291 (p = 0.0086) and 16 h earlier than the animals infected with the 630 wild type strain (p = 0.0184).

The diversity in times to clinical end point observed in the R20291 strain and the incomplete colonisation recorded is most likely a result of the strains sensitivity to the antibiotic clindamycin (MIC = 16 μg/ml) [6]. It has previously been shown that 8 days following administration of a single 30 mg/kg dose of clindamycin, the antibiotic could be isolated at levels of 9 μg/g from the intestinal tract of hamsters. The levels recovered could be sufficient to inhibit colonisation with the sensitive R20291 strain [7].

Here we have shown that the introduction of an *ermB* gene into R20291 confers on the strain created, CRG20291, increased resistance to erythromycin and clindamycin which leads to 100% colonisation in the hamster model and a reduction in the time from infection to clinical end point by 21.8 h compared to R20291. The reproducible colonisation and mortality rates observed with CRG20291 will allow for direct comparison of a “wild type” strain with any ClosTron generated mutants.

## Authorship/contribution

MLK undertook the phenotypic comparison of the strains and the hamster studies with help from MMC and AC, YKN made the vector pMTL-YNC:*ermB* with input from STC, NPM conceived the study and with MLK wrote the manuscript.

## Figures and Tables

**Fig. 1:**
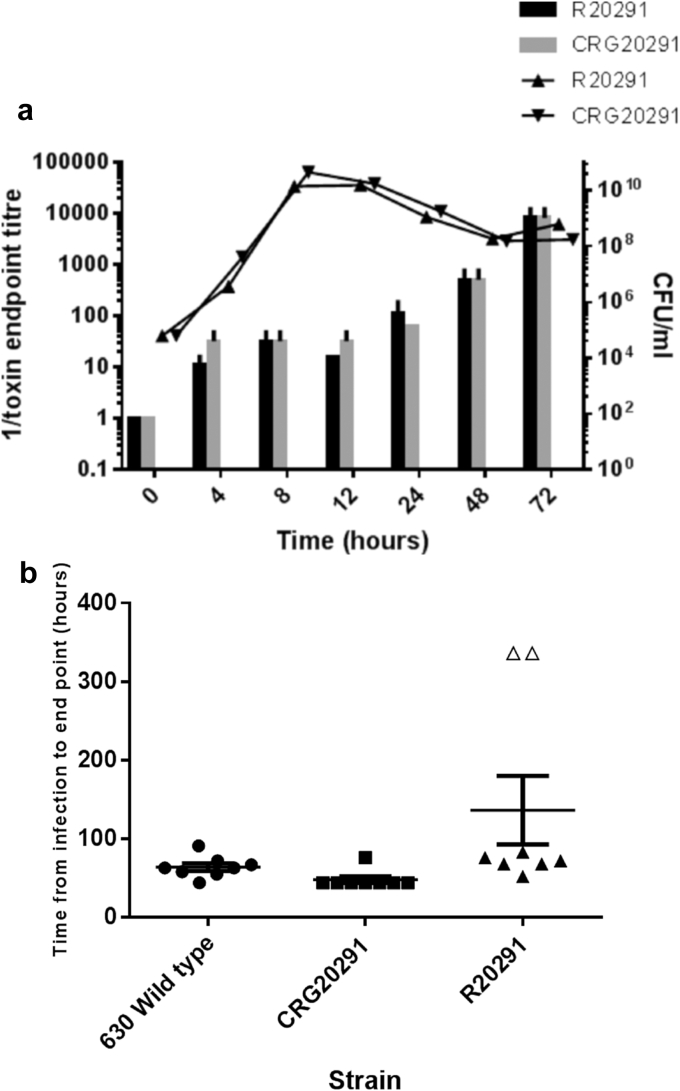
Comparative phenotypic properties of R20291 and CRG20291. **a.** Strains were cultured for 72 h in BHIS and colony forming units (CFU) were determined by serial dilutions and plating **(shaded bars).** The activity of the toxin production in cell culture supernatants was determined by titrating out toxin activity on Vero cells **(triangle shapes). b.** Virulence of *C. difficile* strains in hamsters. Hamsters were challenged with 630 (n = 8), R20291 (n = 8) or R20291M (n = 8). Time from infection to clinical endpoint by each strain is presented in hours. The duration of the experiment was set at 336 h. The open triangles were animals not found to be colonised at the end of the experiment. All statistical analyses were performed using the GraphPad Prism 5 (GraphPad Prism Software). Student *t*-tests were carried out followed by a Mann Whitney test to determine significant difference between groups of animals. *P* values ≤ 0.05 were considered significant.

**Table 1:** Clindamycin MICs for *C. difficile* strains 630, R20291 and CRG20291.

Clindamycin (μg/ml)	*C. difficile* strains			
630 (WT)	630Δerm	R20291	CRG20291
0	++	++	++	++
0.1	++	++	++	++
1	++	++	++	++
10	++	++	++	++
12	++	++	++	++
14	++	++	++	++
16	++	+	+	++
18	++	–	–	++
20	++	–	–	++
30	++	–	–	++
40	+	–	–	++
50	–	–	–	+
80	–	–	–	–
100	–	–	–	–

++ growth observed, + weak growth observed, – no growth observed.
