# Supplementary material for: Improving the reproducibility of the NAP1/B1/027 epidemic strain R20291 in the hamster model of infection
Source: Anaerobe. 2016 Jun;39:51–3. doi: 10.1016/j.anaerobe.2016.02.011 (PMC4879870; doi:10.1016/j.anaerobe.2016.02.011)
Supplement: Supplementary file 1 [file mmc1.docx]

SUPPLEMENTARY INFORMATION

**Improving the reproducibility of the NAP1/B1/027 epidemic *Clostridium difficile* strain R20291 in the hamster model of infection**

M.L. Kelly^1^, Y.K. Ng^1^, S.T. Cartman^1,2^ ^ª^, M.M. Collery^1^, A. Cockayne^1^ and N.P. Minton^1^**^, 2^**.

^1^ Clostridia Research Group, BBSRC/EPSRC Synthetic Biology Research Centre (SBRC), , School of Life Sciences, Centre for Biomolecular Sciences , University of Nottingham, University Park, Nottingham, NG7 2RD, United Kingdom

^2^ Nottingham Digestive Disease Centre, NIHR Biomedical Research Unit, The University Of Nottingham, University Park, Nottingham, United Kingdom.

**EXPERIMENTAL PROCEDURES**

**Creation of an *ermB* derivative of R20291**

Allele-Coupled Exchange (ACE) has previously been used to create a mutant of R20291 which is auxotrophic for uracil due to the precise deletion of 235 bp from the 3’-end of the chromosomal *pyrE* gene. As this defect also renders the cell resistant to 5-fluoroorotate (FOA), its derivation allowed a heterologous *pyrE* gene that restores sensitivity to FOA to be used as a counter-selection marker when incorporated into a specifically fabricated knock-out vector, pMTL-YN4 (Ng et al., 2013). Following the creation of an in-frame deletion of the targeted gene, the design of the *pyrE* deletion is such that the auxotrophic host can be rapidly (2 days) restored to prototrophy using an appropriate *pyrE* ACE correction vector, pMTL-YN2. This allows the phenotype of the deletion mutant made to be characterised in an otherwise clean, wild-type background. When restoring the mutant to uracil prototrophy, the *pyrE* deletion strain may also be simultaneously complemented through incorporation into the ACE complementation vector (pMTL-YN2C, a vector equivalent to pMTL-YN2, but which carries multiple cloning sites downstream of its *pyrE* allele) a wild type copy of the inactivated gene. This becomes inserted into the genome concomitant with restoration of the *pyrE* allele to wild-type (Ng et al., 2013).

The delivery of DNA cargo into the genome by pMTL-YN2C need not be restricted to those genes needed for complementation studies. Any application specific module may be used. For instance, it has recently been used to introduce functional copies of the *C. difficile tcdR* gene downstream of the restored *pyrE* genes of *pyrE* mutants of *Clostridium sporogenes* and *Clostridium acetobutylicum* (Zhang *et al.,* 2015). The strains created may be used, amongst other things, as the host for the previously described *mariner* transposon plasmid pMTL-SC1, where the *mariner* transposase gene utilised is under the transcriptional control of the promoter of the TcdB toxin gene -one of the only two promoters recognised by the TcdR sigma factor. The system, therefore, provides the ideal mechanism for stably inserting a functional copy of a ClosTron-derived *ermB* gene into the *C. difficile* chromosome.

Functional *ermB* genes are derived from the ClosTron when the group II intron and encompassed *ErmB* RAM undergo retrohoming. During this process, the inserted group I intron within the *ermB* gene is self-catalytically removed concomitant with the insertion of the group II intron and, now functionally active, *ermB* gene into the genome.

To create such a strain we synthesised the *ermB* gene of pMTL007C-E1 [10] such that it was immediately preceded by a sequence encompassing a clostridial thiolase gene promoter (P_thl_) and beginning with a NotI restriction site and was followed after its translational stop codon by a HindIII restriction site (see Fig. S1). This appropriately cleaved DNA fragment was cloned between the NotI and HindIII restriction sites of the ACE (Allele-Coupled Exchange, [11]) complementation vector pMTL-YN2C [12] to yield the plasmid pMTL-YN2C::*ermB.* It was transferred into the previously created *pyrE* mutant of R20191 (CRG2358), and transconjugants initially selected on Thiamphenicol supplemented plates. A randomly selected, thiamphenicol resistant transconjugant was thereafter re-streaked onto minimal media lacking uracil. Eight of the putative uracil prototroph colonies that arose were patch plated onto BHIS agar media supplemented with either thiamphenicol or erythromycin. In every case, no growth was evident on the former media, whereas all eight clones grew on media containing erythromycin. Isolated DNA from the clones were PCR screened using the primers that flanked the targeted site of insertion at the *pyrE I* locus (Cdi630-pyrD-SF1 and ermB-HindIII-R) and shown to generate a fragment of the expected size (1549bp). Sanger sequencing of the fragment generated confirmed that insertion of *ermB* had occurred at the intended position in the chromosome (Fig. S2). One random clone was selected for further study and designated CRG20291.

**Cell growth.**

Strains were cultured in Brain Heart Infusion (Oxoid) (BHIs) broth to measure cell growth and production of toxin A and toxin B over a 72h time-course. Each *C. difficile* strain was cultured from storage at -80^0^C by streaking onto BHIs medium. Single colonies were picked into 1ml of BHIs medium and incubated overnight. The overnight culture was then diluted 1 in 100 into fresh BHIs medium to start the assay. Colony forming units were determined at 0, 4, 8, 12, 24, 48 and 72 h by making serial dilutions and plating onto BHIs agar. At the same time-points, toxin A and toxin B activity were measured by cell cytotoxicity assay, as described below.

**Vero and HT29 cell cytotoxicity assays.**

Vero (African green monkey kidney) and HT29 (Human colon carcinoma) cell monolayers were prepared by seeding each well of a 96 well plate with 100µl of cell suspension at a density of 2x 10^5^ cells/ml. Vero cells and HT29 cells were cultured in DMEM and McCoy’s 5A, respectively, supplemented with 10% v/v foetal calf serum and 1% v/v penicillin/streptomycin (Invitrogen). Plates were incubated for 48 h (37^0^C, 5% CO_2_) to let monolayers form before addition of *C. difficile* culture supernatants. *C. difficile* culture supernatants were harvested by centrifugation (12,000 x g for 2 min) and filtration (0.2 µm pore-size). Four- fold serial dilutions were made in PBS and 20 µl of each was added separately to the 100 µl of medium above Vero and HT29 cell monolayers. After 24 h incubation (37^0^C, 5% CO_2_), monolayers were examined by light microscopy (Nikon Eclipse TS100) to determine the toxin end-point titer for each supernatant sample. End-point titer was defined as the first dilution in a series where call morphology was indistinguishable from the negative controls. Results were expressed as ‘1/toxin end-point titer’.

**Clindamycin sensitivity test.**

Brain Heart Infusion agar (Oxoid) (BHIs) was prepared and supplemented with clindamycin hydrochloride (Sigma) in a range of concentrations from 0 – 100 µg/ml. Each *C. difficile* strain was cultured from storage at -80^0^C by streaking onto BHIs medium. Single colonies were then picked and re-streaked on to BHIs medium supplemented with clindamycin. Plates were incubated for 24 hours and the Minimum Inhibitory Concentration (MIC) was designated as the lowest concentration where growth was inhibited.

**Spore preparation.**

Strains were incubated for 5 days on Brain Heart Infusion agar (Oxoid) supplemented with 0.5% yeast extract and 0.01% L-cysteine (Sigma) (BHIs) under anerobic conditions. Following incubation all growth was harvested and re-suspended in 1 ml PBS. Suspensions were heat treated at 65^0^C for 30 minutes; the samples were pelleted by centrifugation (12,000 x g for 1 min). Pellets were washed in PBS for a total of three times. Following the final wash step the pellets were re-suspended in dH_2_O. Spores were enumerated by plating onto BHIs

**Animal Experiments.**

All animal procedures were carried out in accordance with the United Kingdom Home Office Inspectorate under the Animals (Scientific Procedures) Act 1986. All experiments were approved by the University of Nottingham Ethics committee and carried out under licence number 40/29761. A block design with final group sizes of 8 was used for this study.

Female Golden Syrian hamsters of between 100 – 120g were obtained from Charles River U.K. All animals were housed individually in individual ventilated cages (IVCs)and were given free access to food and water. Each animal was given clindamycin hydrochloride (30mg/kg) via the oral route five days prior to infection orally with 10,000 spores of the 630 wild type strain, the R20291 strain or the CRG20291 CRG2932 strain. Following infection animals were monitored for signs of infection and euthanised when a pre-determined end point was reached. Faecal pellets were collected daily and upon euthanasia a caecum sample was taken from each animal and stored in 1ml PBS.

**Treatment of faecal and caecum samples.**

Samples were homogenised using a Precelly 24 bead homogeniser, followed by centrifugation (4,600 x g for 1 minute). The supernatants were removed and heat treated at 65^0^C for 30 minutes. Samples were plated onto Clostridium difficile agar base supplemented with D-cycloserine (0.25 mg/ml), Cefoxitin (0.008 mg/ml), 0.1% Taurocholic acid and Ampheterian (2.5 mg/ml) (Sigma). Faecal samples were incubated for 24 hours to track colonisation.

Following incubation of caecum samples single colonies which appeared to share morphology with *C. difficile* were picked and re-streaked on to fresh plates. Single colonies were then picked into 1ml fresh BHIs media and incubated overnight prior to DNA extraction.

**PCR conformation of *C. difficile* strains.**

To confirm the *C. difficile* isolated from the caecum samples was the same strain originally used during infection, DNA was isolated from the overnight cultures. PCR amplification was carried out using primer pairs specific to each host; 630 wild type (4140 and 5880R), R20291 (CDSM0-239-F1 and CDSM0-239-R1) and CRG2932 (Cdi-630-pyrD-sF1 and ermB-HindIII-R) (Table S2).

**Statistical analysis.**

All statistical analyses were performed using the GraphPad Prism 5 (GraphPad Prism Software). Student t tests were carried out followed by a Mann Whitney test to determine significant difference between groups of animals. *P* values ≤0.05 were considered significant.

**Figure S1. The Nucleotide sequence of the *ermB* gene and thiolase gene promoter**

NotI -35 P_thl_  -10

GCGGCCGCGAATTGCGTTATATTGATAAAAATAATAATAGTGGGTATAATTAAGTTGTTAGA

GAAAACGTATAAATT**AGGAGG**GATTGAT**ATG**AACAAAAATATAAAATATTCTCAAAACTTTTTAACGAGTGAAAAAGTACTCAACCAAATAATAAAACAATTGAATTTAAAAGAAACCGATACCGTTTACGAAATTGGAACAGGTAAAGGGCATTTAACGACGAAACTGGCTAAAATAAGTAAACAGGTAACGTCTATTGAATTAGACAGTCATCTATTCAACTTATCGTCAGAAAAATTAAAACTGAATACTCGTGTCACTTTAATTCACCAAGATATTCTACAGTTTCAATTCCCTAACAAACAGAGGTATAAAATTGTTGGGAGTATTCCTTACCATTTAAGCACACAAATTATTAAAAAAGTGGTTTTTGAAAGCCATGCGTCTGACATCTATCTGATTGTTGAAGAAGGATTCTACAAGCGTACCTTGGATATTCACCGAACACTAGGGTTGCTCTTGCACACTCAAGTCTCGATTCAGCAATTGCTTAAGCTGCCAGCGGAATGCTTTCATCCTAAACCAAAAGTAAACAGTGTCTTAATAAAACTTACCCGCCATACCACAGATGTTCCAGATAAATATTGGAAGCTATATACGTACTTTGTTTCAAAATGGGTCAATCGAGAATATCGTCAACTGTTTACTAAAAATCAGTTTCATCAAGCAATGAAACACGCCAAAGTAAACAATTTAAGTACCGTTACTTATGAGCAAGTATTGTCTATTTTTAATAGTTATCTATTATTTAACGGGAGGAAA**TAA**AAGCTT

HindIII

The position of the NotI and HindIII restriction sites have been underlined and labelled. The ATG start codon of the *ermB* gene and its TAA stop codon have been emboldened and underline. The position of the ribosome binding site of *ermB* has also been emboldened.

**Figure S2. Genetic arrangement of the *C. difficile* chromosome showing the position of insertion of P_thl_ and *ermB***

**
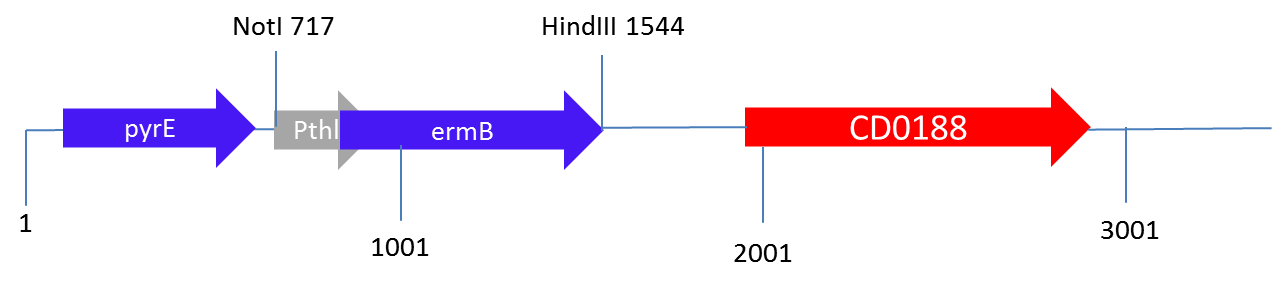
**

**TABLE S1 Strains and Plasmids used in this study**

| **Strains/Plasmids** | **Relevant features** | **Source** |
| --- | --- | --- |
| *C. difficile* R20291 wild type | BI/NAP1/027 Stoke Mandeville (2004-2005) isolate | Val Hall, Anaerobic Reference Centre, Cardiff |
| *C. difficile* 630 wild type | First *C. difficile* strain to have its genome determined | Peter Mullany, UCL |
| CRG2358 | *C. difficile* R20291 Δ*pyrE* | Ng *et al.,* 2013 |
| CRG2932 | *C. difficile* R20291:*ermB* | This Study |
| pMTL-SC2 | Used as a template for *ermB* gene | This Study |
| pMTL-YN2C | ACE correction vector with multiple cloning site | Ng *et al.,* 2013 |
| pMTL-YN2C::ermB | ACE correction vector containing the *ermB* gene and thiolase gene promoter | This Study |

**TABLE S2 Oligonucleotide Primers used in this study**

| **Oligonucleotide** | **Sequence (5’ to 3’)** |
| --- | --- |
| 4140 | TAAGAGTGTGTTGATAGTGC |
| 5880R | GTCAAGTAAGCAAACATAGTCG |
| CDSMO-239-F1 | GCTATTATTATGCCAGGATACTTTTATACACC |
| CDSMO-239-R1 | GACTCACTAATTTCTATTCCATATATTGATGC |
| Cdi-630-pyrD-sF1 | TAGAGAAGGAATAAAAAGTTTAGACGAAATAAGAGG |
| ermB-HindIII-R | AAAAAAAAGCTTTTATTTCCTCCCGTTAAATAATAGATAACTATTAAAAATAG |
